# Infrequent and Potentially Missed Cause of Hypoxemia in an Infant

**DOI:** 10.7759/cureus.5766

**Published:** 2019-09-25

**Authors:** Elham A Elgabaly, Ajay P Dsouza, Aji Mathew, Muhammad Anwar

**Affiliations:** 1 Pediatric Radiology, Al Jalila Children Specialty Hospital, Dubai, ARE; 2 Radiology, Al Jalila Children Specialty Hospital, Dubai, ARE; 3 Pediatric Pulmonology, Al Jalila Children Specialty Hospital, Dubai, ARE

**Keywords:** childhood interstitial lung disease (child), high-resolution computed tomography (hrct) of lungs, ground glass opacities

## Abstract

Neuroendocrine cell hyperplasia of infancy (NEHI) is a recently reported condition and commonly missed. A general pediatrician who encounters an infant with an insidious onset of breathlessness, hypoxemia, and failure to thrive should think through a diagnosis of NEHI when common respiratory diseases are excluded. Lung biopsy is regarded as the diagnostic gold standard for NEHI and typically demonstrates increased numbers of neuroendocrine cells (NECs) in otherwise near-normal lung tissues. However, classic high-resolution computed tomography (HRCT) findings can enable to establish the diagnosis without the need for a biopsy. This case shows typical chest imaging findings of NEHI with a brief review of the literature.

## Introduction

Neuroendocrine cell hyperplasia of infancy (NEHI) is a rare childhood interstitial lung disease (chILD), which presents within the first two years of life with common but challenging key clinical features. The clinical presentation is usually nonspecific, The child initially presents with persistent tachypnea, crackles, and hypoxemia without fever. The patients are usually treated as either chest infection or reactive airway disease without significant improvement on usual treatment. The disease presents with chronic and recurrent symptoms leading to failure to thrive. Increased awareness among pediatrician of the clinical presentation may enable timely diagnosis and improve disease management and prognosis.

We report a case of NEHI, presenting with chronic and recurrent episodes of shortness of breath and failure to thrive. The infant had many prior admissions at a number of hospitals and was treated one time as a chest infection and another time as a reactive airway disease. The diagnosis of NEHI was initially suggested by imaging findings in X-rays and subsequently confirmed by high-resolution computed tomography (HRCT) of the chest.

## Case presentation

An eight-month-old infant presented to our institution with a four-month history of recurrent episodes of cough and dyspnea without fever, associated with failure to thrive, requiring repeated hospital admissions. The antenatal and perinatal course was unremarkable.

During the repeated hospital admissions, diagnoses of chest infection and/or reactive airway disease were presumed, but the child did not respond to antibiotics or bronchodilators. Eight months old, the child presented to us with respiratory failure. He had tachypnea, chest retraction, and severe desaturation (SpO_2_ 54 mmHg). There was no fever, and blood counts were normal with negative inflammatory markers. Developmental delay and failure to thrive were also noted.

**Figure 1:**
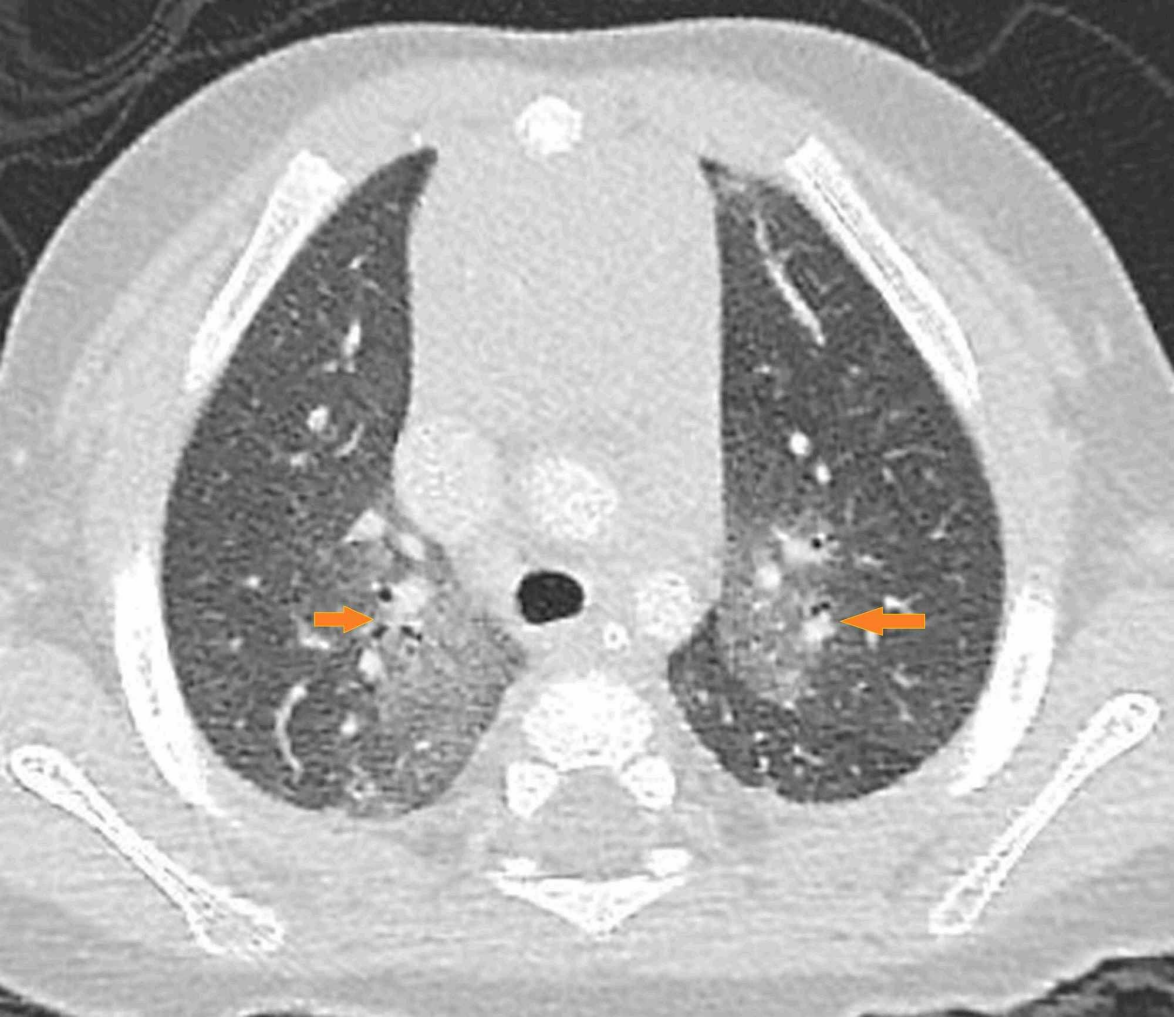
Axial HRCT section of the lung at the level of the upper lobe Arrows on both sides show the geographic ground-glass opacities. HRCT, high-resolution computed tomography

**Figure 2:**
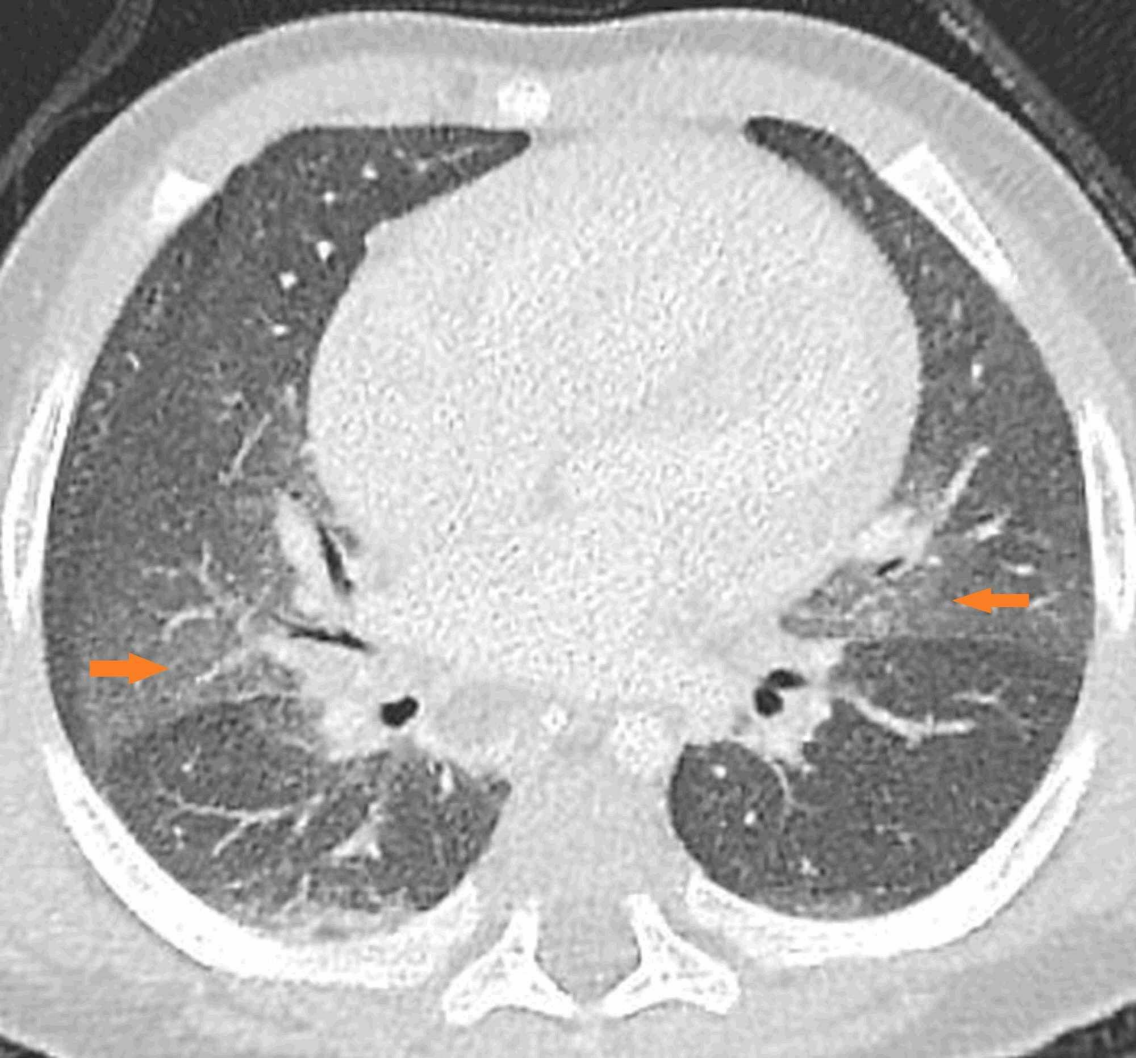
Axial HRCT section of the lung at the level of the middle lobe Arrows on both sides show the geographic ground-glass opacities. HRCT, high-resolution computed tomography

**Figure 3:**
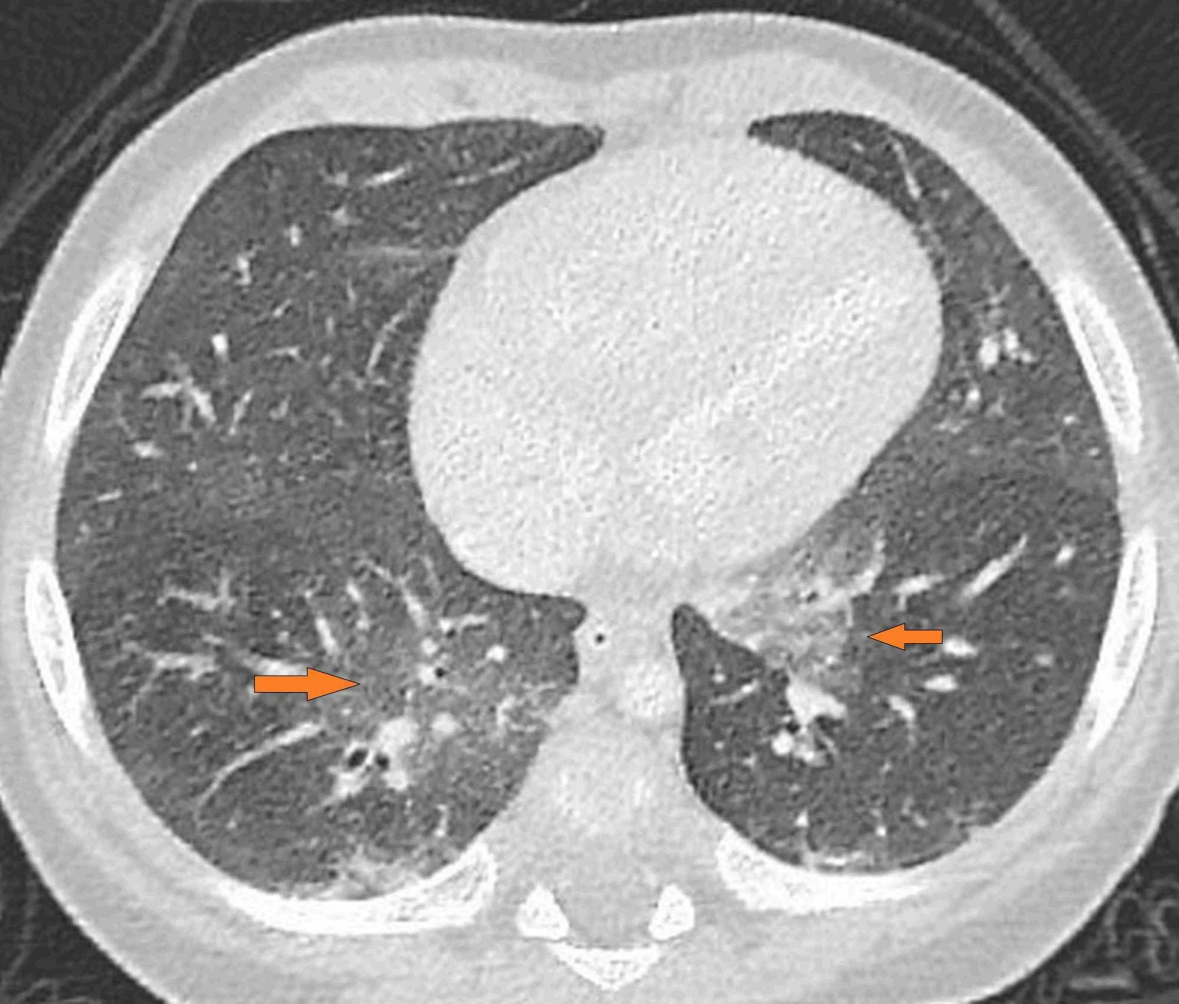
Axial HRCT section of the lung at the level of the lower lobe Arrows on both sides show the geographic ground-glass opacities. HRCT, high-resolution computed tomography

The sweat test for cystic fibrosis was negative and echocardiography was normal. Oxygen supplementation improved saturation to 94%, but chest retraction and tachypnea persisted. Chest X-rays showed bilateral symmetrical hyperinflation of lungs with “fluffy” perihilar infiltrates; these findings were potentially consistent with either chest infection or small airway inflammation. An HRCT scan of the lung was performed, which showed the characteristic findings of NEHI as seen in the images below (Figures 1-3). The child clinically improved on oxygen supplementation and was discharged on home oxygen therapy and nutritional supplementation.

 

The clinical presentation suggests the following differential diagnosis: Bronchiolitis obliterans, NEHI, pulmonary edema, and pulmonary alveolar proteinosis.

We conclude that this case is consistent with NEHI, because the patient presents clinically with recurrent episodes of cough and dyspnea without fever, not responding to antibiotics, plus the characteristic HRCT appearance of bilateral geographic ground-glass opacities in the centri-bronchovascular distribution in the right middle lobe and lingula (Figures 1-3), air-trapping and hyperinflation in the basal lung with no other pleuroparenchymal or airway abnormality detected.

## Discussion

chILD is a group of uncommon disorders categorized by abnormal imaging, altered gas exchange leading to significant illness [1]. Children who meet at least three of the following four criteria are classified as having chILD [2]:

1. Respiratory symptoms of a cough, tachypnea, or exercise intolerance [3],

2. Clinical signs of resting tachypnea,

3. Adventitial sounds, chest retractions, digital clubbing,

4. Failure to thrive or respiratory failure,

5. Documentation of hypoxemia, and

6. Diffuse lung abnormalities on chest imaging [1].

NEHI is a form of chILD first reported in 2005 as persistent tachypnea of infancy [3]. NEHI is a unique sub-group of chILD, which is more prevalent in infants and children younger than two years [4]. NEHI manifests in the first year of life as gradual-onset persistent tachypnea, chest retractions, hypoxemia, and failure to thrive. The diagnosis of NEHI is challenging as the symptoms are nonspecific and masquerade as common respiratory illnesses of infancy and childhood, requiring a systematic approach [1].

NEHI is rare, and the cause is unknown. The incidence and prevalence are also unknown [2]. Genetic predisposition is also postulated as familial cases have been found [2]. When a child presents with chronic respiratory symptoms like tachypnea, recurrent cough, exercise intolerance, and failure to thrive, evaluation and exclusion of more common diseases are required before making a diagnosis of NEHI. Acute and chronic Infection, asthma, congenital heart disease, immunodeficiency, and cystic fibrosis are more common than NEHI.

Chest radiography may be non-specific and may show hyperinflation with peribronchial thickening, mimicking viral lower respiratory illness or reactive airway disease. When an initial workup is unrevealing, and the more common conditions are excluded, a further workup for childhood interstitial lung disease should begin. HRCT of the chest is the study of choice [5]. 

HRCT findings in infants with NEHI are characteristic and specific. In an appropriate clinical setting, HRCT lung establishes the diagnosis. The findings are geographic ground-glass opacities in the centri-bronchovascular distribution in the right middle lobe and lingula (Figures 1-3). Air-trapping and hyperinflation in the basal lung are also noted. No other pleuroparenchymal or airway abnormality is seen. In the correct clinical context, the specificity of HRCT for the diagnosis of NEHI approaches 100 percent, thus, avoiding an invasive lung biopsy [2]. 

No definitive treatment is available for NEHI; general and supportive measures include optimizing nutrition, immunizations, treatment of intercurrent illness, and prevention of inhalation of cigarette smoke and other inhaled irritants are recommended. Supplemental oxygen is required [6].

## Conclusions

NEHI is a non-progressive chILD insidiously presenting in infancy. Its recognition is important as it can go undiagnosed and can be mistaken for common respiratory illness in this age group thus delaying diagnosis and leading to ineffective treatment. A high index of suspicion is needed as the child undergoes clinical and imaging evaluation. Increased awareness among pediatricians and timely diagnosis improves outcomes and prevents unnecessary investigations. Children who meet the chILD criteria should be subjected to HRCT lungs to confirm the diagnosis, thus avoiding invasive diagnostic methods.
